# Adaptations in the Treatment of Congenital Lymphedema Centered on the Quality of Life

**DOI:** 10.1155/2014/456060

**Published:** 2014-02-17

**Authors:** Jose Maria Pereira de Godoy, Ana Paula Sanchez, Daniel Zucchi Libanore, Maria de Fatima Guerreiro Godoy

**Affiliations:** ^1^Cardiology and Cardiovascular Surgery, Department of the Medicine School in São José do Rio Preto (FAMERP), 15025-120 São Jose do Rio Preto, SP, Brazil; ^2^Research Group in Godoy Clinic, 15025-120 São Jose do Rio Preto, SP, Brazil; ^3^Medicine School in São José do Rio Preto (FAMERP), Godoy Clinic, 15025-120 São Jose do Rio Preto, SP, Brazil

## Abstract

*Case Description*. This report describes the evolution, necessary adaptations, and complications in the treatment of a 9-year-old child with primary congenital lymphedema. *Description of Intervention*. The clinical treatment of lymphedema was started in the first year of the patient's life and for five years she was only treated using the Godoy & Godoy technique of cervical stimulation. Three years ago the patient was prescribed a compression stocking made from a cotton-polyester fabric (grosgrain) because of a sudden increase in the lymphedema after she started to take growth hormones. *Outcome and Conclusion*. The combination of cervical stimulation and a compression stocking was effective to keep the child's life relatively normal, performing all day-to-day and recreational activities.

## 1. Introduction

Lymphedema is defined as the abnormal buildup of protein-rich fluid in soft tissues resulting from dysfunction of the lymphatic system, that is, an imbalance between the formation of lymph and its absorption into the initial lymphatic system [1].

Primary lymphedema is classified as idiopathic or family (hereditary). Hereditary lymphedema may or may not be associated with congenital anomalies or physical changes [2]. It has great clinical heterogeneity, both within and between families and the age at onset varies. There are more than four distinct genetically known conditions related to lymphedema with mutations of three genes having been discovered in families with lymphedema [3].

The recommended treatment for lymphedema is a combination of therapies including manual and mechanical lymph drainage, compression (garments and bandages), myolymphokinetic exercises and activities, and hygienic care [4–9].

## 2. Case Report

**Figure 1:**
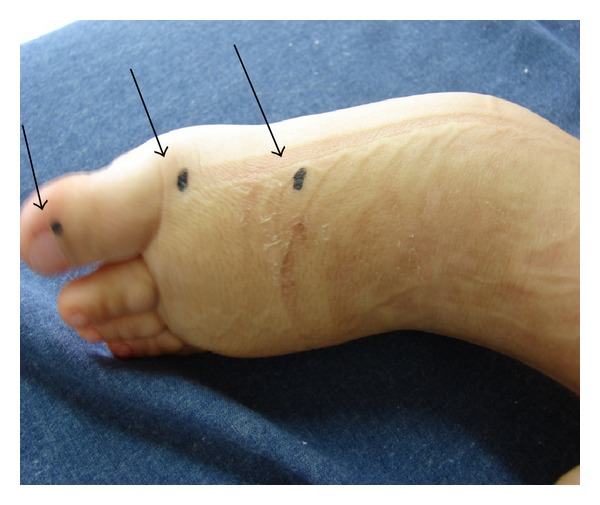
Points of measurement of the perimetry of the foot.

**Figure 2:**
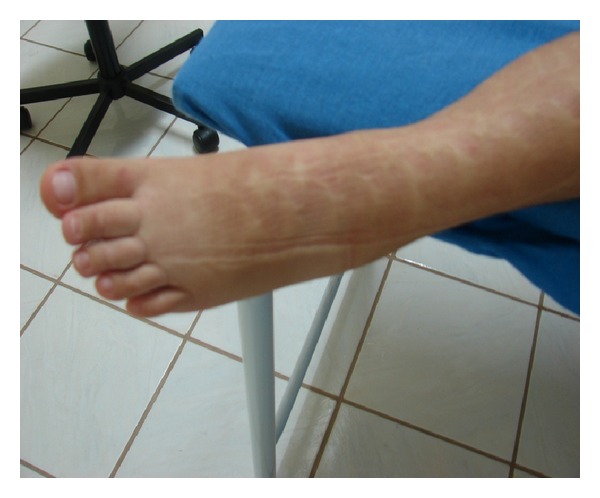
Normalization of the edema using an association of a grosgrain compression stocking and cervical stimulation.

The case of a nine-year-old female child, who was born with congenital lymphedema of the left leg, is reported. She was referred for treatment when she was one year old. Diagnosis of lymphedema was clinical with perimetric measurements (every 3 cm) along the foot and leg (Figure 1). Cervical stimulation using the Godoy & Godoy technique was performed until the age of six. It was initially carried out by the medical team but, after teaching the mother, she performed the cervical stimulation with clinical control being carried out by the medical team initially every week, then every fortnight, and eventually once per month. Cervical stimulation is the only lymph drainage technique that has been evaluated in isolation and proven to be efficacious in the reduction of edema. The hypothesis of the mechanism of action is that cervical stimulation causes the contraction of the lymphangions thereby increasing drainage [10]. Cervical stimulation as monotherapy reduced the perimetry of the limb to within the clinically normal size range without signs of edema in the early morning when the child got up within around three months. At the age of six, a diagnosis of growth hormone deficiency was reached and hormone therapy was initiated. The child began to grow quickly with aggravation of the lymphedema with an increase of 3 cm in the perimetry of the dorsum of the foot. A hand-made low-stretch compression stocking of a cotton-polyester fabric (grosgrain) was added to the treatment regimen. This fabric has low stretch across the material and elasticity in the other direction thus allowing good flexibility of the limb but providing compression. The association of compression to the treatment reduced and controlled the edema. The patient, now nine years old, has been receiving hormones for three years with her leg remaining within the normal size range compared to the contralateral limb. Normalization of the edema using an association of a grosgrain compression stocking and cervical stimulation occurred in about three months (Figure 2).

When the child wanted to do ballet classes, the mother was advised not to allow her due to repetitive movements involved and the exertion of the foot. As the child insisted, she was permitted and encouraged to use a cotton-polyester compression stocking; changes in the size of the leg were monitored every week, clinically and by perimetry. There were no variations in the size of the leg and the child took ballet classes for more than one year.

This study was approved by the Research Ethics Committee of FAMERP (number 3822010).

## 3. Discussion

This case report describes the developments in the clinical treatment of a patient with primary congenital lymphedema. Only one form of therapy, cervical stimulation as proposed by Godoy & Godoy was used until the age of six (see video in [10]) [10–13]. The hypothesis of the mechanism of action of this stimulation is that it stimulates contractions of lymphangions thereby physiologically assisting lymph drainage. This treatment has been used to treat lymphedema in general, either alone or associated to other types of therapy [11, 12].

A combination of therapies is recommended for the treatment of lymphedema because of the synergistic effect produced with greater reduction in the swelling. The primary option for children is cervical stimulation as this technique improves the lymphedema and is easy to perform; even the mother or members of the family can be trained to perform the procedure. The time spent should normally be between 15 to 20 minutes in one session and can be carried out while the child is sleeping. Using this technique, the family is able to carry out treatment independently. During the period that the mother is undergoing training she must be given guidance on the care necessary such as precautions related to infection, injuries to the leg, and the type of activities the child can do. Primarily the mother is advised to let the child have as near to normal life as possible but to look out for possible health problems. If worsening of the lymphedema is noticed, the mother is told to report it immediately to the physician for him to try to identify the problem.

The proposal is to use the minimum intervention necessary to control the edema. The association of the compression stocking in this patient was because of the rapid increase in the size of the foot in the first month after being prescribed growth hormone. The hypothesis for the worsening of the edema during hormone therapy is the acceleration in growth resulting from the medication. Today the patient is 9 years old and has been receiving growth hormone for three years; however, the size of the leg is maintained within the normal size range using compression therapy with a grosgrain stocking and cervical stimulation. This association has a synergistic effect in reducing edema [13]. The child has accepted the use of the stocking until now without intolerance or psychological changes. However, when additional forms of therapy such as the compression stocking are used, other problems may be created. In this case, the stocking could have caused problems in relation to the child's embarrassment with her classmates; luckily with this patient, this did not happen.

Mechanical and manual lymph drainage, which are other forms of treatment in lymphedema, were not used in this case as cervical stimulation and compression stockings are forms of treatment that basically only require the involvement of the family and thus give a certain independence from the medical team. Other therapies, such as manual lymph drainage, would have been used if required during the course of treatment.

The greatest care is needed in respect to infections, mainly erysipelas, that may aggravate the lymphedema and so guidance on this should be constantly given during the treatment program. Monthly control is fundamental to identify problems and follows the development of the child. She showed no skin changes that could negatively impact on the treatment. Counseling on evaluating the foot every day in order to identify injuries or mycosis is critical to prevent infection, chiefly erysipelas. Adjustments to the stocking are also essential; if it is incorrectly used, it will just be uncomfortable for the patient without giving any benefits. In the latter part of the treatment of this patient, the use of the stocking was the only way by which a reduction, normalization, and control of the edema were achieved.

Daily activities that require much force or that involve long periods of sitting or standing without movement of the limb may aggravate the edema. Activities that require much force of the muscles require a greater amount of blood, which, in turn, requires greater capillary filtration but also causes external compression of the vessel providing a propelling function [14, 15]. There should be a balance between capillary filtration and the amount drained if not this can lead to edema and worsening the lymphedema. Thus the family received guidance on the type of activities that may aggravate the edema and the necessary cares.

Simple and effective treatment was adapted for this patient so as not to excessively affect her quality of life and so that she was able to evolve as a normal child. Ballet classes were permitted, but extra supervision was provided. It is important to remember that not all activities should be allowed; some must be adapted and some prohibited. Creativity, involving the medical team and the family, is crucial for the adaptation of this approach to treatment. In conclusion, the individual adaptation of therapy in the treatment of lymphedema can be efficient and provide greater family independence.
